# Supplementary material for: Brd2 haploinsufficiency extends lifespan and healthspan in C57B6/J mice
Source: PLoS One. 2020 Jun 19;15(6):e0234910. doi: 10.1371/journal.pone.0234910 (PMC7304595; doi:10.1371/journal.pone.0234910)

**Supplementary Figure 1:** qPCR analysis of tissue and MEFs from WT and *Brd2*+/- mice measuring the levels of *Brd2* transcript.


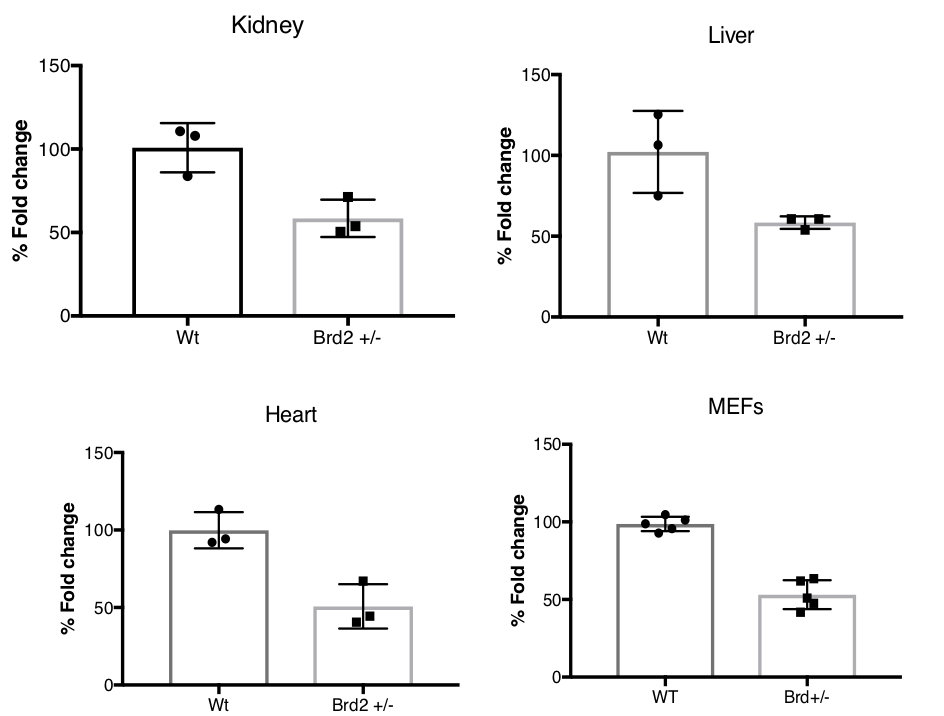

Supplement: S1 Fig — (DOCX) [file pone.0234910.s001.docx]
