# Supplementary material for: Brd2 haploinsufficiency extends lifespan and healthspan in C57B6/J mice
Source: PLoS One. 2020 Jun 19;15(6):e0234910. doi: 10.1371/journal.pone.0234910 (PMC7304595; doi:10.1371/journal.pone.0234910)

**Supplementary Figure 2:**

Although it is well known that caloric restriction is associated with increased lifespan in C57B6/J mice, we do not see any evidence in support of body weight differences in our HET and WT mice.


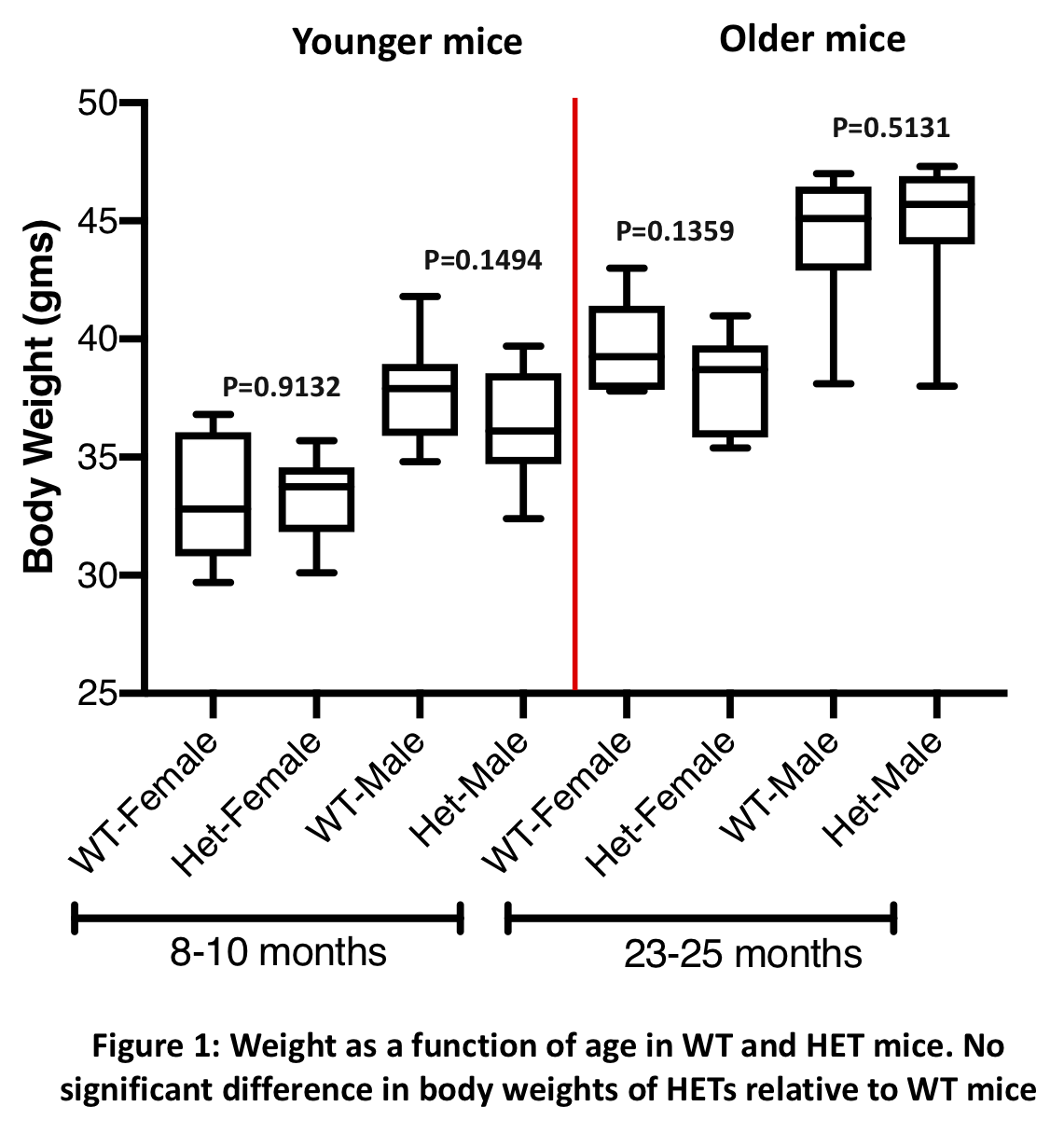

Supplement: S2 Fig — (DOCX) [file pone.0234910.s002.docx]
