# Supplementary material for: Brd2 haploinsufficiency extends lifespan and healthspan in C57B6/J mice
Source: PLoS One. 2020 Jun 19;15(6):e0234910. doi: 10.1371/journal.pone.0234910 (PMC7304595; doi:10.1371/journal.pone.0234910)
Supplement: S3 Fig — (A & B): Hematoxylin & Eosin staining in WT liver (A) showing inflammation (thin black arrows) and more macrovascular vacuolation (thick, black arrow) in WT than HET (B). (C & D) Hematoxylin & Eosin staining in WT spleen (C) and HET(B) showing disorganization of the splenic structure in WT. (E & F) Hematoxylin & Eosin staining in WT (A) and HET(B) testes showing increased vacuolation in WT (C) as compared to HETs (D). (DOCX) [file pone.0234910.s003.docx]

**Supplementary Figure 3:**


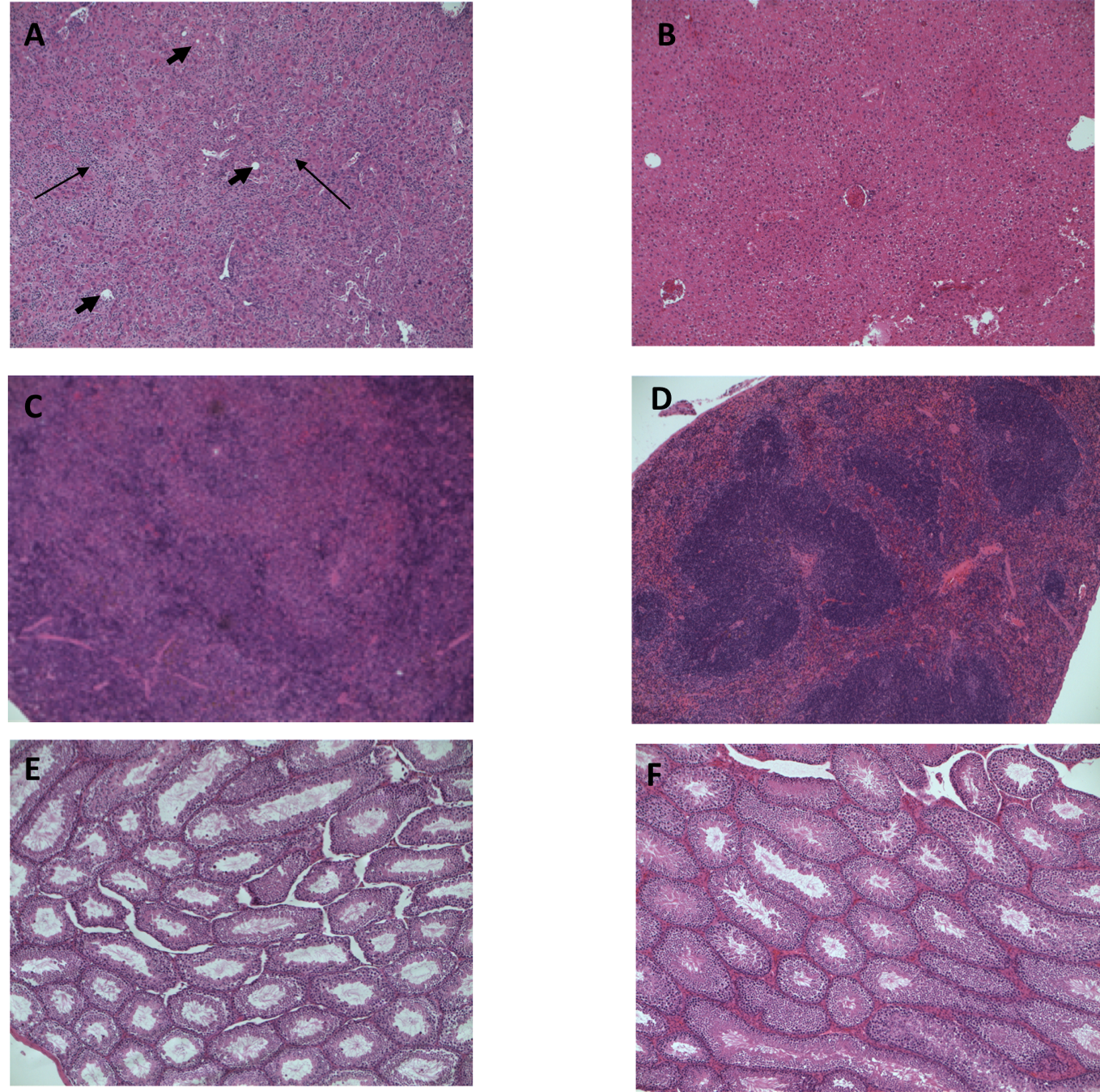
Histological differences in representative liver, spleen and testis sections from age matched WT and HET mice (n=6) at 18 months. (A & B): Hematoxylin & Eosin staining in WT liver (A) showing inflammation (thin black arrows) and more macrovascular vacuolation (thick, black arrow) in WT than HET (B). (C & D) Hematoxylin & Eosin staining in WT spleen (C) and HET(B) showing disorganization of the splenic structure in WT. (E & F) Hematoxylin & Eosin staining in WT (A) and HET(B) testes showing increased vacuolation in WT (C) as compared to HETs (D).
