# Supplementary material for: Brd2 haploinsufficiency extends lifespan and healthspan in C57B6/J mice
Source: PLoS One. 2020 Jun 19;15(6):e0234910. doi: 10.1371/journal.pone.0234910 (PMC7304595; doi:10.1371/journal.pone.0234910)
Supplement: S1 Raw images — (DOCX) [file pone.0234910.s005.docx]

Figure 3F (Upper Panel): Immunoblot showing p53 expressed from WT and HET livers.


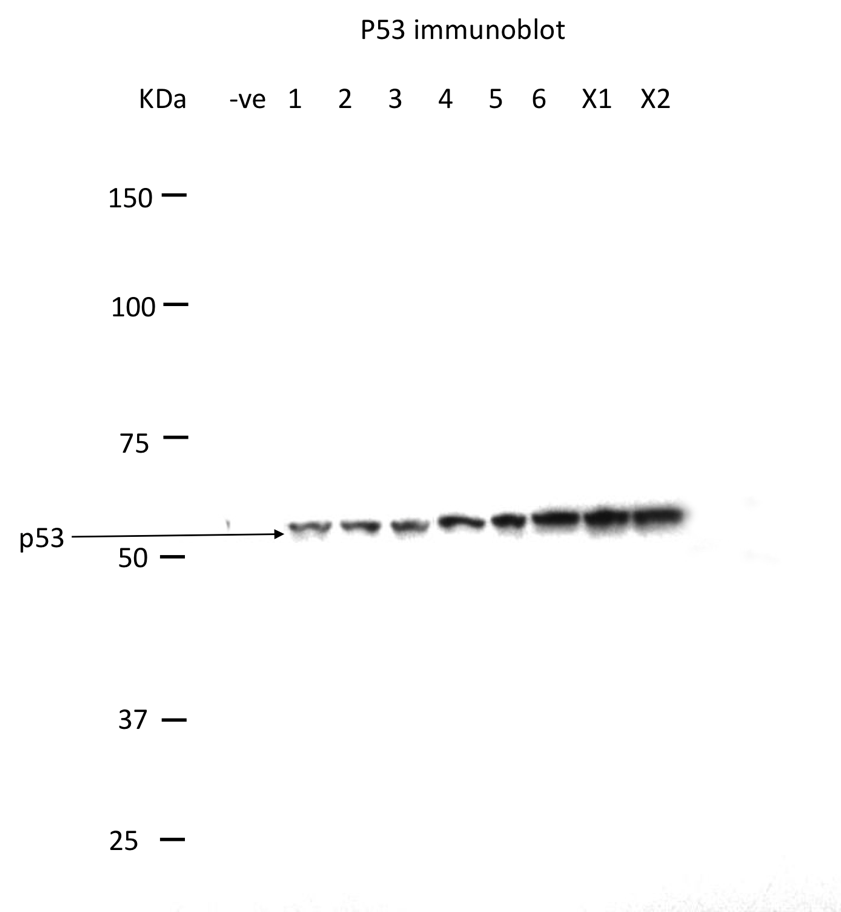


Lane -ve: No primary antibody (anti-p53) negative control

Lane 1, 2, 3: WT liver

Lane 4,5,6: Het liver

Lane X1, X2: Not included in the final image

The immunoblots were developed on a Typhoon FLA 9500 imager. The final figure panel was generated using lanes 1,2,3,4,5, and 6.

Figure 3F (Lower Panel): Immunoblot showing Gapdh expressed from WT and HET livers.


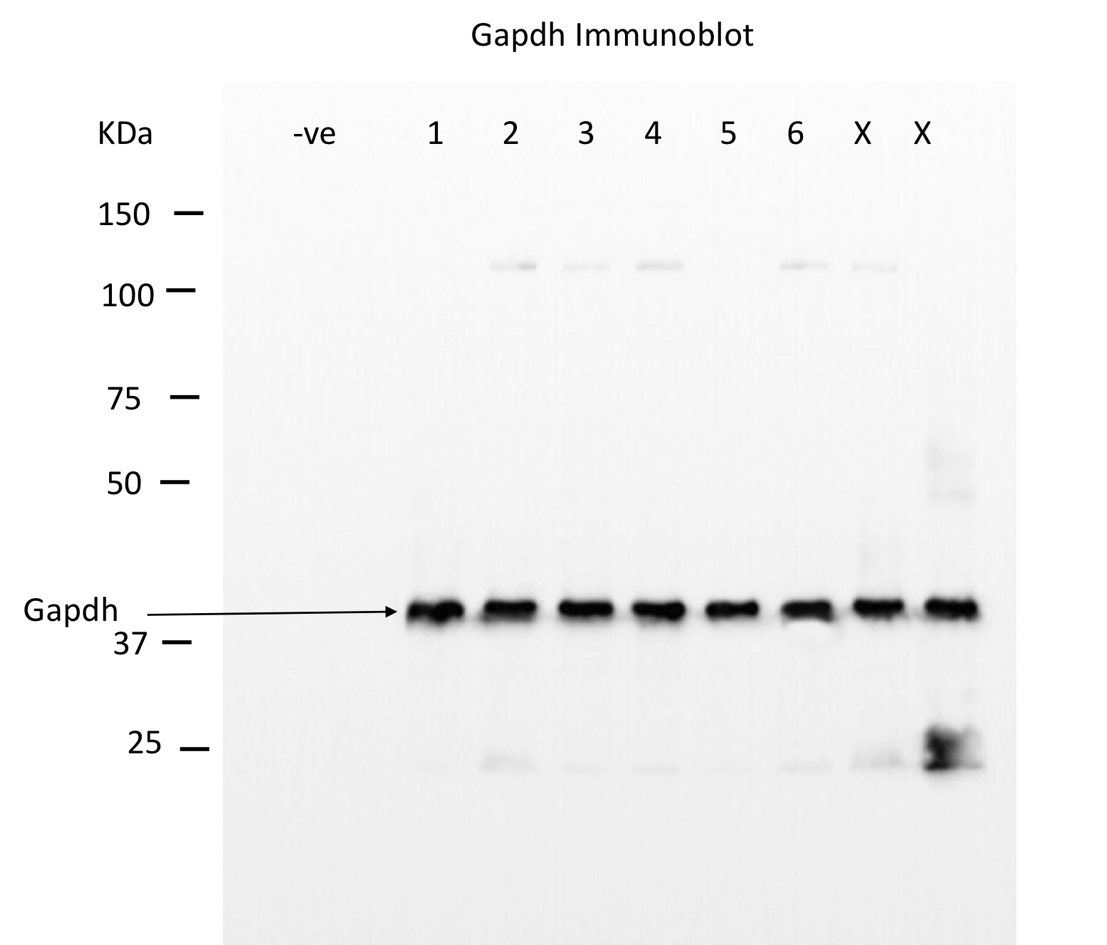


Lane -ve: No primary antibody (anti-Gapdh) negative control

Lane 1, 2, 3: WT liver

Lane 4,5,6: Het liver

Lane X1, X2: Not included in the final image

The immunoblots were developed on a Typhoon FLA 9500 imager. The final figure panel was generated using lanes 1,2,3,4,5, and 6.
